# The Latest Trends and Evolution of HIV Medication Management: A Systematic Review

**DOI:** 10.7759/cureus.91933

**Published:** 2025-09-09

**Authors:** Charity Iheagwara, Stanley Ezulike, Mishame Anja, Okelue E Okobi

**Affiliations:** 1 Infectious Diseases, Saint Michael's Medical Center, Newark, USA; 2 Internal Medicine, Chukwuemeka Odumegwu Ojukwu University Teaching Hospital, Awka, NGA; 3 Medicine, Blacklion Specialized Hospital, Addis Ababa, ETH; 4 Family Medicine, Larkin Community Hospital Palm Springs Campus, Miami, USA; 5 Family Medicine, IMG Research Academy and Consulting LLC, Homestead, USA

**Keywords:** antiretroviral therapy, drug resistance, hiv infections, long-acting injectable antiretrovirals, medication adherence

## Abstract

The last 10 years have witnessed various significant progresses in the management and treatment of HIV/AIDS, including the development of novel HIV prevention technologies and safer and potent antiretroviral medications. Therefore, this systematic review aims to evaluate and summarize some of the recent trends in HIV medication management. To attain the stated objective, we conducted an in-depth literature search on virtual medical databases including PubMed, Medline, Scopus, and Google Scholar for peer-reviewed articles highlighting novel findings on HIV medication trends. The findings of this systematic review have disclosed that the comprehension of the HIV lifecycle was a breakthrough that enabled the development of effective targeted medications. Further, advancements in prevention interventions have resulted in HIV becoming an increasingly manageable condition. Thus, the amalgamation of biomedical, structural, and behavioral approaches has also reduced the infection rates, even as the progress toward the realization of an effective vaccine continues to sustain the hope of realizing a HIV-free globe. Thus, regardless of the prolonged battle against HIV/AIDS, extant evidence indicates that bringing the epidemic to an end is probable and within our grasp.

## Introduction and background

Forty years after the initial reporting of Pneumocystis jirovecci and Kaposi’s sarcoma clusters in New York- and Los Angeles-based homosexuals, HIV/AIDS is still a global public health challenge and concern [1]. According to the recent statistics (2023), approximately 39.9 million individuals are living with HIV/AIDS globally, even as 1.3 million people are projected to have contracted the disease in 2023, and with an average mortality rate of 6.2 per 100,000 individuals in the same year [2]. Consequently, a larger proportion of individuals living with HIV/AIDS are residents of low- and middle-income countries (LMICs), with the sub-Saharan Africa region being the most affected, with approximately 20.8 million individuals living with the disease as of 2023 [3]. As such, the concerted efforts made in the last four decades have resulted in human immunodeficiency virus infections moving from horrific and lethal diseases to increasingly manageable conditions [4]. For instance, combination antiretroviral therapy (ART) has been effective in significantly reducing the viral load to undetectable levels (<50 RNA copies/mL), leading to a considerable prevention of opportunistic infections and reconstitution of the immune systems of persons living with HIV [5]. As a result, many individuals living with HIV are presently living normal lives.

Nevertheless, regardless of the above attainments, the HIV pandemic still affects women disproportionately, particularly in nations and regions with prevalent other transmission routes [1-4]. Females have limited alternatives to safeguard themselves from HIV infections, and approaches aimed at promoting abstinence, condom use, and monogamy have not been effective in preventing the spread of the disease and are impractical in most contexts [1-3]. Thus, women face significant challenges in efforts to convince their male partners to be faithful, monogamous, or use condoms. Consequently, female condoms that were developed to enable women to have more control in HIV prevention and protect themselves have not been widely accepted, even as their higher costs and structural concerns have hindered their adoption. However, HIV pre-exposure prophylaxis (PrEP) is offering a promising novel approach to reducing HIV spread across the globe [6]. Still, the development of various microbicides, comprising products that are mostly applied in the vagina and rectum for protection against HIV, offers increased potential for women-controlled preventive alternatives that do not require any consent, control, or knowledge of one’s partner [7-10]. Although microbicides have the potential of being beneficial to women and men, their effective usage is still reliant on their approval and efficiency. Long-term success in the management and treatment of HIV requires continuous advancement and improvement of existing medications, as well as the development of newer drugs with fewer side effects, prolonged action with lasting virologic suppression, and a higher genetic barrier to resistance development [11-15].

Notably, being an RNA virus, HIV converts the RNA into double-stranded DNA upon entry into the human cell, with the aid of the reverse transcriptase (RT), which was the main target of the initial HIV medications, including zidovudine (AZT) [3]. The FDA developed and approved various nucleoside reverse transcriptase inhibitors (NRTIs) in 1991, including didanosine, zalcitabine, and stavudine [16-18]. Regrettably, the RT enzyme is highly prone to errors, and HIV rapidly develops mutants capable of escaping such medications, leading to resistance to drugs and drastic relapse in patients. The introduction of protease inhibitors in the 1990s marked the start of the development of highly active antiretroviral therapies (HAART), leading to a significant reduction in mortality rates. Subsequent innovations, which included the integrase inhibitors and long-acting injectables, have enhanced adherence, effectiveness, and tolerability. Currently, PrEP and other new drug formulations have continued to improve HIV prevention, management, and treatment, marking significant scientific advancement. As a result, HIV medication and management have evolved and transformed the condition from a severe and fatal infection to an increasingly manageable condition. Thus, since the discovery of HIV in the 1980s, significant progress has been realized with ART, particularly the progression from single-drug treatments with higher toxicity levels and increased resistance risks to innovative combination therapies. Therefore, this systematic review sought to evaluate the latest trends and evolution in HIV medication management, particularly with a focus on advancements in medication research efforts aimed at different viral enzymes and cellular host factors that have led to the advancements in HIV medication management.

## Review

Materials and methods

This systematic review involved the performance of an in-depth and exhaustive literature search on various electronic databases, including PubMed, Scopus, Web of Science, Embase, and Google Scholar for studies published between 2015 and 2025. The selected studies for this review included multicenter studies, health assessment studies, prospective cohort studies, epidemiological studies, and systematic reviews. Duplicate data sources were mainly identified by comparing studies with similar population years. Further, different MeSH keywords were utilized in the literature search, including HIV infections, antiretroviral therapy, medication adherence, drug resistance, and long-acting injectable antiretrovirals. The in-depth literature search conducted resulted in a total of 186 articles.

Inclusion and exclusion criteria

After removing the duplicate studies, relevant studies were selected based on a three-phase procedure. The initial phase involved the screening of the studies’ titles and abstracts, while the second phase involved the exclusion of all irrelevant studies. The final phase involved the performance of a comprehensive full-text assessment of all references to verify their significance. Consequently, three independent reviewers were tasked with screening the references, and possible disagreements were resolved through consensus and discussions.

For this study, the inclusion criteria targeted original studies, including randomized controlled trials (RCTs), prospective cohort studies, and crossover studies, among others that satisfied the following set criteria: original studies published in peer-reviewed journals, studies published between 2015 and 2025, full-text articles, and studies originally published in the English language. Furthermore, to be included, the studies had to focus on assessing the latest trends in HIV medication management. Conversely, the exclusion criteria included expert editorials, opinion pieces, narrative reviews, studies without relevance to the target populations, and sponsored clinical trials. Further, irrelevant and inaccessible studies and those with irrational methodologies were excluded. Additionally, for this study, the data extracted from included studies consisted of the general attributes of the study, such as the sampling methods used, author names, and publication years; demographic attributes, such as sample size, age, sex, race/ethnicity, and follow-ups; as well as data on interventions and durations. The major findings of every study were systematically documented.

Results

**Figure 1:**
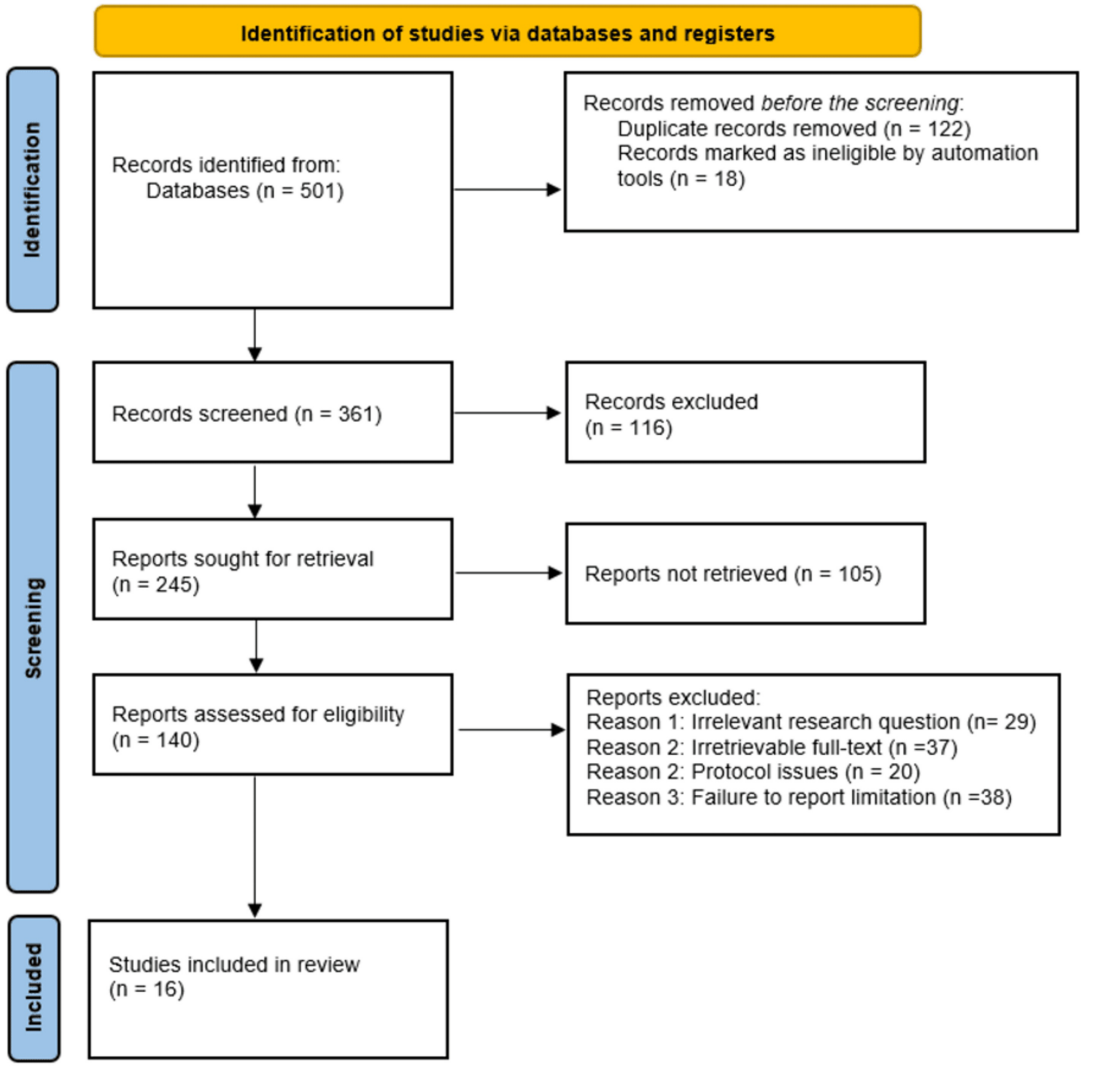
Preferred Reporting Items for Systematic Reviews and Meta-Analyses (PRISMA) flow diagram showing the study selection and inclusion process for this systematic review. n: Number

This study utilized the Preferred Reporting Items for Systematic Reviews and Meta-Analyses (PRISMA) guidelines in the selection and inclusion of pertinent literature, with the initial in-depth database search yielding 501 references. Following the first screening of the studies, 122 duplicates were excluded, alongside 18 references that were marked ineligible by automation. The remaining 361 studies were subjected to screening of titles and abstracts, which led to the exclusion of 116 studies due to factors that included irrelevance to the study topic, as the abstracts showed divergent objectives from the study. Following the screening, 245 studies were sought for retrieval, out of which 105 studies were not retrieved. This led to 140 studies being assessed for eligibility, which led to the exclusion of 130 studies for various reasons, including irrelevant research questions (29), irretrievable full-text (37), protocol issues (20), and failure to report limitations (38). Eventually, 16 studies met the inclusion criteria and were subsequently included in this study. These studies were then assessed and discussed together with the findings of other relevant studies that corroborate this study’s findings [19-39]. The PRISMA flow diagram outlining the article selection process for this study is shown in Figure 1 below.

Quality assessment

The quality of the included studies was assessed using AXIS, a 20-item critical appraisal tool for cross-sectional studies [9]. Three independent reviewers were tasked with assessing each study and resolving potential disagreements through discussions and consensus. The responses were also scored as 1 (yes), 0 (no), or “don’t know” for inapplicable items. In general, a larger proportion of the included studies were rated high quality, with only two rated as moderate. Table 1 below shows the outcomes of the quality assessment of the included studies conducted using the AXIS critical appraisal tool.

**Table 1:** Results of the quality assessment of the included studies conducted using the AXIS critical appraisal tool. ✓: Yes

Study/Citation	Study Design	Clear Objectives?	Appropriate Methodology?	Data Collection Rigor?	Risk of Bias?	Are the results clearly reported?	Applicability to the research question?	Overall quality
Gandhi et al. [19]	Expert consensus guidelines	✓	✓	✓	Low	✓	High	High
Corado et al. [20]	Narrative review	✓	Limited systematic methods	Moderate	Moderate	✓	Moderate	Moderate
Gaikwad et al. [22]	Methodological review	✓	✓	✓	Low	✓	High	High
Tseng et al. [23]	Historical review	✓	Narrative focus	Moderate	Moderate	✓	Moderate	Moderate
Dorward et al. [27]	Policy analysis	✓	✓	✓	Low	✓	High	High
Menéndez-Arias et al. [29]	Systematic review	✓	✓	✓	Low	✓	High	High
Taki et al. [30]	Drug review	✓	✓	Limited comparative data	Moderate	✓	Moderate	Moderate
Agrahari et al. [31]	Technology review	✓	✓	✓	Low	✓	High	High
Mayer et al. [32]	RCT (DISCOVER trial)	✓	✓	✓	Low	✓	High	High
Prather and Jeon [33]	Drug review	✓	✓	No new data	Moderate	✓	Moderate	Moderate
Carr et al. [34]	Clinical perspective	✓	✓	✓	Low	✓	High	High
Dubey [35]	Short review	Lacks depth	Moderate	Moderate	High	Moderately	Low	Low
Hitchcock et al. [36]	Drug review	✓	✓	✓	Low	✓	High	High
Hussein et al. [37]	Basic science review	✓	✓	✓	Low	✓	High	High
Matsuda and Maeda [38]	Basic science review	✓	✓	✓	Low	✓	High	High
Schwarzer et al. [39]	Conceptual review	✓	✓	Theoretical	Moderate	✓	Moderate	Moderate

Table 2 below indicates the summary of the various studies and guidelines selected and included in this systematic review.

**Table 2:** Summaries of the studies and guidelines included in this systematic review. ART: antiretroviral therapy, PrEP: pre-exposure prophylaxis, AZT: zidovudine, STRs: single-tablet regimens, TAF: tenofovir alafenamide, FTC: emtricitabine, TDF: tenofovir disoproxil fumarate, LMICs: low- and middle-income countries

Guideline/Study/Citation	Key Findings
Gandhi et al. [19]	Updated IAS-USA 2024 guidelines recommend integrase strand transfer inhibitors (INSTIs), including dolutegravir/bictegravir, as first-line ART. Places emphasis on long-acting injectable, including cabotegravir/rilpivirine, for maintenance therapy and highlights PrEP advancements.
Corado et al. [20]	Two-drug regimens, including dolutegravir/lamivudine, portrayed efficacy in treatment-naïve and maintenance therapy, reducing side effects and pill burden while also maintaining virologic suppression.
Gaikwad et al. [22]	Reviewed the cell-based assays for anti-HIV drug screening, stressing high-throughput methods for evaluation of viral entry, reverse transcriptase, and integrase inhibition.
Tseng et al. [23]	Assessed the trajectory of ART from toxic early regimens (AZT) to safer, simpler therapies (INSTIs, STRs). The study has also discussed challenges that include drug resistance and aging-related comorbidities.
Dorward et al. [27]	The study found dolutegravir (DTG) to be cost-effective for LMICs but raises concerns regarding weight gain and neural tube defects in pregnancy.
Menéndez-Arias and Delgado [29]	The study focused on the latest ART classes (capsid inhibitors, broadly neutralizing antibodies) and various strategies for latency reversal and immune modulation.
Taki et al. [30]	The study disclosed that cabotegravir/rilpivirine (CAB/RPV), as the first long-acting injectable ART (every 2 months), showed higher efficacy but requires adherence to the injection schedules.
Agrahari et al. [31]	Reviews long-acting PrEP technologies (e.g., injectable cabotegravir, implants, nanoparticles) and challenges like drug release kinetics and cost.
Mayer et al. [32]	The DISCOVER trial: TAF/FTC PrEP non-inferior to TDF/FTC, with enhanced bone/renal safety but higher weight gain.
Prather and Jeon [33]	The study disclosed that cabotegravir injectable PrEP (every 2 months) reduced HIV risk by 69% compared to oral TDF/FTC, even though it requires clinic visits, and also has injection-site reactions.
Carr et al. [34]	The study disclosed that the rising INSTI resistance (including dolutegravir) underscored the need for resistance testing, as well as new agents like lenacapavir.
Dubey [35]	The study found that long-acting injectable (CAB/RPV, lenacapavir) improved adherence despite having several barriers, including the requirement for cold-chain storage and high cost.
Hitchcock et al. [36]	The study found that lenacapavir, a twice-yearly capsid inhibitor, is effective in multidrug-resistant HIV, with an exceptional resistance profile.
Hussein et al. [37]	CRISPR-Cas9 demonstrated a promise in excising HIV proviral DNA despite facing significant delivery challenges and off-target risks.
Matsuda and Maeda [38]	The study reviewed "shock-and-kill" (latency reversal) and "block-and-lock" (permanent silencing) strategies for HIV reservoirs.
Schwarzer et al. [39]	The study has proposed combining latency reversal with immune enhancers, including checkpoint inhibitors, for sustained ART-free remission.

Data extraction

The authors utilized a data extraction form to extract relevant data from included studies. Data about the different characteristics of the studies, such as the studies’ general attributes, author names, and publication year; demographic attributes, including sample size, gender, age, follow-ups, and race; as well as the interventions utilized, intervention duration, and measurement methods, were extracted. This was followed by the systematic recording of the key study findings.

Discussion

In the 1980s, HIV patients had an average life expectancy of about one year. Currently, timely and early initiation into ART has resulted in persons living with HIV (PLWH) expecting near-normal lifespans. However, drug resistance alongside side effects has driven the ongoing need for new drugs. Though initially developed in 1964 as a cancer medication, AZT reintroduction as a potential treatment for HIV offered a breakthrough, offering a ray of hope for the successful treatment and management of HIV [10]. Thus, AZT successfully suppressed HIV replication without causing any damage to cells, leading to clinical trials aimed at evaluating the medication in PLWH by Burroughs Wellcome (Durham, NC, USA) [11]. Used alone, AZT resulted in a significant decrease in HIV deaths and opportunistic infections, although with severe adverse side effects. Despite its various benefits, AZT also presented limitations that prompted additional research. AZT belongs to the NRTI class of antiretroviral medication. As a result of the discovery, AIDS Clinical Trials Group (CTG) commenced to build on the drug, with the ACTG 016 clinical trial lowering the AZT treatment dosage, which aided in the reduction of some of the adverse side effects of AZT. Consequently, the crucial ACTG 019 clinical trial assessed whether there were any advantages in placing PLWH on AZT treatment before their conditions progressed to AIDS [12]. The study disclosed that AZT was effective in delaying AIDS onset in asymptomatic PLWH, marking the initial demonstration of successful HIV infection treatment [8,12]. 

The single-drug therapies’ limitation also became increasingly clear, given the ability of HIV to replicate faster and the liability of errors during the replication. Such errors and mutations often result in changes in the virus [10]. Moreover, HIV variants with mutations conferring resistance to antiretroviral medications can drastically evolve [13]. For instance, in certain individuals who were placed on AZT only, drug resistance was noted in a few days following treatment initiation [14]. Owing to such resistance, HIV researchers tested whether a combination of different medications would make it challenging for the virus to be simultaneously resistant to the medications. As a result, AZT and dideoxycytidine (ddC)/zalcitabine were administered jointly to PLWH in the early 1990s and indicated that the two-drug treatment was more effective compared to AZT only, thereby raising hopes regarding the utilization of combination therapy in HIV treatment [15]. Clinical trials also portrayed the superiority of combination therapies. For instance, the outcomes of the ACTG 175 trial conducted in 1995 indicated that two-drug combination treatment was increasingly superior to AZT only in the prevention of death and CD4+ cell count decline [16]. The trial results disclosed that ART was effective in reducing the risk of mortality in persons with asymptomatic and intermediate-phase disease. Still, the Community Programs for Clinical Research on AIDS (CPCRA 007) trial evaluated the effectiveness of combination therapy for individuals with advanced-stage HIV, most of whom had been previously placed on AZT [17]. The CPCRA 007 trial disclosed that two-drug therapy did not have any significant benefit over AZT only with regard to slowing the disease progression and mortality in the patient group [18]. Nevertheless, among the CPCRA 007 participants with minimal or no previous use of AZT, combination therapy proved more effective than AZT only. The ACTG 175 and CPCRA 007 trials' outcomes, alongside those of other studies, demonstrated that previous antiretroviral use might significantly affect the effectiveness of certain treatments and medications, highlighting the significance of cautious planning with regard to medication management and use of ARVs in HIV treatment. Owing to such observed limitations of two-drug HIV treatments, long-lasting HIV suppression was sought using triple-drug therapy. Notably, the strategy involving the use of two NRTIs alongside a potent third drug is still the cornerstone of existing treatment principles and is referred to as combination antiretroviral therapy [19]. Tenofovir alafenamide (TAF), tenofovir disoproxil fumarate (TDF), lamivudine (3TC), and emtricitabine (FTC) are still the recommended nucleoside and nucleotide reverse transcriptase inhibitor components of the first ART regimen [19].

Although two-NRTI drug therapy proved better compared to single-drug therapy for many PLWH, their duration was limited [19,20]. As a result, significant advancements were realized in 1996, when clinical trials disclosed that triple-drug therapies could durably suppress the replication of HIV to minimal levels, even as they created a higher genetic barrier against drug resistance development [21]. The triple-drug therapy’s probability and success, known as HAART, was partly as a result of the appearance of a novel ARV medication class, protease inhibitors [22]. Thus, saquinavir was the initial protease inhibitor to get US FDA approval in late 1995, even as the results of a trial conducted in 1996 disclosed that a triple-drug therapy consisting of AZT, saquinavir, and ddC was increasingly effective compared to a two-drug therapy with AZT and ddC [21-24]. Among the studies that have indicated increased effectiveness of triple-drug therapy is the ACTG 320 trial, which disclosed that adding two novel drugs during the switching of therapy was increasingly effective compared to adding a single novel drug [25]. Notably, with HAART, which entails a combination of drugs from two or more distinct classes, several PLWH experienced a significant drop in the level of HIV in their blood to undetectable levels [22,24]. Despite the observation that HAART was a lifesaver, the initial treatment regimens were imperfect, given the complexity of daily dosing and burdensome side effects. Specific medications were, as a result, administered in combination at diverse intervals throughout the day, some requiring food while others did not need food. Such complexities made it increasingly challenging for PLWH to adhere to the treatment regimens in the long term. 

Advancements in drug development resulted in increasingly manageable treatments. Thus, to effectively tackle the complexities associated with ARV regimens, drug resistance, and drug toxicities, various studies have been carried out, some ongoing, to formulate and develop new medications capable of working through diverse mechanisms and targeting the different stages of the HIV replication process [26]. At present, over 30 ARV medications are in existence, including numerous fixed-dose combinations consisting of two or more medications from different drug classes in one tablet. Currently, PLWH can manage their condition by taking only one tablet a day [22,26]. Nonetheless, the mid-1990s witnessed the emergence of a novel class of ARV medications known as the non-nucleoside reverse transcriptase inhibitors (NNRTIs). Given their inexpensive and easier production processes compared to the protease inhibitors, NNRTIs have significantly assisted in scaling up ARV treatments within resource-restricted contexts [8,23]. Still, the identification of new drug targets has been pivotal in the discovery and subsequent development of novel ARV drug classes. For instance, since the 1980s, HIV researchers have acknowledged that the CD4 molecule is the main HIV receptor on the immune cells [22]. As such, the National Institute of Allergy and Infectious Diseases (NIAID) researcher disclosed the discovery of the C-X-C chemokine receptor type 4 (CXCR4) co-receptor, needed by specific HIV strains to enter into immune cells. The discovery resulted in the pursuit of the co-receptors by HIV researchers. Thus, various research groups and organizations, including NIAID researchers, established the existence of another receptor, C-C chemokine receptor type 5 (CCR5), which is the main co-receptor utilized by HIV in infecting immune cells [22]. The discovery of CCR5 formed the basis for the development of maraviroc, a CCR5-blocking medication that was approved by the US FDA in 2007. 

Similarly, 2007 witnessed the development of another key ARV drug class, following the integrase inhibitor raltegravir's approval by the FDA [22-26]. Thus, raltegravir fast turned out as an important component in combination antiretroviral therapies; however, HIV might follow numerous conduits in developing resistance to the medication. Further, HIV variants resistant to raltegravir might additionally prove resistant to elvitegravir, a different first-generation integrase inhibitor. The 2013 FDA approval of dolutegravir, a second-generation integrase inhibitor with a higher barrier to HIV drug resistance development, was a success, as clinical trials found the drug effective in PLWH who had not been initiated into any HIV therapy and in treatment-experienced PLWH, including individuals for whom the first-generation integrase inhibitors had been ineffective. Dolutegravir was also beneficial as it had a higher safety profile, was a convenient once-daily dosage, and had a comparatively lower cost of production. At present, the U.S Department of Health and Human Services has recommended the inclusion of dolutegravir in two of the first-line regimens, particularly for adult PLWH, even as the World Health Organization guidelines recommend its use as an alternative first-line medication for adults [27].

Consequently, it is noteworthy that HIV medication management has witnessed significant advancements with regard to effectiveness, prevention, tolerability, and convenience, from 2013 to the present. For instance, there have been significant improvements in ART regimens, including the introduction of single-tablet regimens (STRs). Thus, before 2013, HIV treatments utilized multi-pill regimens; however, this has changed, and STRs are presently the gold standard, enhancing medication adherence [21-26]. Traditionally, medication management of HIV has entailed a combination of three or more ARV drugs. However, recent advancements have resulted in a growing shift to regimens offering similar effectiveness with one or fewer medications. Such simplification has reduced the pill burden in addition to lowering the side effects risks and aids in preserving prospective treatment alternatives through limiting drug resistance [24,26]. Currently, the dolutegravir/lamivudine (Dovato) regimen is widely prescribed for the treatment and management of HIV in naïve patients with no drug resistance [24-26]. The long-term efficacy of such single drug regimens has been supported by various studies and clinical data [24,26], even as novel combinations are being introduced, further expanding the existing array of simplified alternatives. One of the major STRs and single drug regimens was Stribild (elvitegravir/cobicistat/emtricitabine/tenofovir DF), which was approved by the US FDA in 2013 [28]. Stribild contains four active ingredients, namely: cobicistat, tenofovir disoproxil fumarate, elvitegravir, and emtricitabine, and is also a complete HIV treatment regimen, implying that it should not be taken alongside other HIV medications to avoid adverse drug interactions [28,29]. Further, Stribild is mainly prescribed for adults and adolescents (12 years and above, weighing a minimum of 35 kg) who have just been initiated into HIV treatment, and with a virus that is not resistant to the drugs making up Stribild [28].

Consequently, between 2015 and 2021, three key medications for the treatment and management of HIV were developed, including Genvoya, Biktarvy, and Cabenuva [29]. Thus, in 2015, Genvoya, a single-dose regimen combining four distinctive antiretrovirals, namely, elvitegravir, cobicistat, emtricitabine, and TAF, replaced Stribild, owing to its safety profile, as it offered better bone and kidney protection [28,29]. As a complete regimen, Genvoya is not used with other HIV drugs, and is taken orally, once daily, and with food. The drug is recommended for adults and children aged two years and above, weighing a minimum of 14 kilograms, and with HIV infection and no resistance mutations to the integrase inhibitor class, tenofovir and emtricitabine [28,29]. Genvoya is also recommended for PLWH without any history of having been initiated into ART or virologically suppressed individuals on stable regimens [28]. Still, in 2018, Biktarvy, which is an integrase strand transfer inhibitor comprising three key antiretrovirals, namely, TAF, bictegravir, and emtricitabine, was approved by the US FDA and turned out as a key STR owing to its attributes that include increased efficacy, requirement for no booster, and minimal side effects [29]. Biktarvy is also a complete regimen for HIV treatment and is not used alongside other HIV medications. The drug has a higher barrier to resistance, implying that Biktarvy is less liable to lose its efficiency as a result of the development of resistance by the virus [29]. Biktarvy is taken orally as a single tablet daily, and the dosage for children may be adjusted based on aspects such as weight. 

Further, Cabenuva (cabotegravir and rilpivirine) was initially approved by the FDA in 2021 as the first long-acting injectable medication (monthly or every two months), and has replaced HIV treatment using daily pills in certain PLWH [30]. Cabenuva has provided a groundbreaking approach concerning the management of HIV infection as a complete prescription regimen developed for PLWH aged 12 years and above, weighing more than 35 kilograms, who have realized virologic suppression (HIV-1 RNA <50 copies/mL), and without prior treatment failures and resistance to either cabotegravir or rilpivirine. Cabenuva is also an effective alternative to extant HIV medications, particularly in instances where clinicians have confirmed that the patient has met the set criteria, ensuring an effortless transition to the innovative treatment and therapy [30]. The offers increased flexibility in administration, enabling an alternative 28-day oral lead-in treatment or a direct-to-injection method, tailored to meet the needs and preferences of the patient while ascertaining convenience and efficacy in the long-term management of HIV [30]. Such advances in HIV medication management represent a key step towards simplification of treatment regimens and the improvement of care and quality of life for PLWH. 

Besides the treatment innovations, improvements in HIV prevention have also been transformative. Notable advancements have also been realized with regard to HIV prevention. According to Agrahari et al., the HIV prevention, treatment, and management landscape has witnessed significant transformation in the last decade, characterized by the groundbreaking advancements in PrEP medications and newer drug classes [31]. For instance, in 2012, the FDA approved Truvada (TDF/FTC) as the initial PrEP regimen, despite its widespread adoption occurring after 2013 [32]. The PrEP alternatives expansion occurred in 2019 following the FDA approval of Descovy (FTC/TAF), even as the drug’s use was restricted to specific populations owing to inadequate study data of its efficacy for cisgender women [32]. Nonetheless, a significant breakthrough was realized in 2021 following the FDA approval and introduction of Apretude (cabotegravir), which is the initial long-acting injectable PrEP, providing an option to the everyday oral medication regimens and effectively tackling the adherence challenge [33]. Additionally, PrEP is an important advancement in HIV prevention, offering effective protection for HIV-negative individuals, particularly those in serodiscordant heterosexual relationships. When taken consistently, PrEP can reduce the risk of transmission of HIV significantly, making it a powerful tool in modern HIV medication management. Integrating PrEP into healthcare systems requires regular HIV testing, adherence support, and ongoing medical monitoring to ensure its safe and effective use. When combined with antiretroviral therapy for HIV-positive partners, PrEP plays a key role in a broader prevention strategy capable of reducing new infections at a population level. Ultimately, PrEP is not just a medication, but a cornerstone of HIV prevention that empowers individuals and strengthens public health responses [6]. Currently FDA approved forms of PrEP, daily pills (Truvada and Descovy), and extended-release injectable suspension (cabotegravir) have made it easier to adhere to PrEP.

Apart from prevention, various innovative drug classes have been developed and have subsequently revolutionized HIV treatment and management, especially in intricate cases. For instance, in 2020, Trogarzo (ibalizumab), an attachment inhibitor, offered a lifeline for persons with multidrug-resistant HIV, offered through bi-weekly intravenous (IV) infusions [34]. Subsequently, in 2022, lenacapavir (Sunlenca) came out as a groundbreaking bi-yearly subcutaneous injectable, drastically reducing the treatment burden in heavily experienced PLWH [34]. Jointly, these advancements are reflective of the dynamic shift towards increasingly accessible, patient-centered, and long-lasting treatment alternatives. Presently, lenacapavir is being evaluated as a potential foundation for future HIV treatments to offer long-acting injectable and oral alternatives with diverse dosing frequencies, as a mono agent or in combination, to tackle the various individual preferences and needs, as well as those of the affected persons and communities [34]. 

Building on such innovations, research has significantly expanded to include a wider array of long-acting therapies. Several studies have also highlighted various latest trends and advancements in HIV medication management. For instance, according to Dubey, amongst the notable and recent developments in HIV medication management is the ongoing evolution of long-acting injectable antiretroviral therapies (LA-ARVs) [35]. Such medications have been noted to provide the necessary alternatives for PLWH experiencing daily pill burden and those seeking increased discretion during care. As of 2025, focus has been placed on LA-ARVs, including cabotegravir and rilpivirine (Cabenuva), which has been approved in many nations and is currently administered on a single-dose per month basis [30]. Various clinical trials are ongoing to assess longer dosing intervals, with possible extension of three to four months, to minimize the requirement for regular clinic visits and enhance patient adherence. Additionally, novel long-acting formulations are in process, and these include lenacapavir, an FDA-approved capsid inhibitor used in the treatment of highly treatment-experienced individuals [36]. Clinical trials using lanacapavir have shown significant promise in instances where the drug is used jointly with other long-acting regimen agents that might be administered once every six months [36]. Such developments are representative of the shift towards making HIV management increasingly flexible and less intrusive, which might aid in reducing stigma and supporting long-term viral suppression. Additionally, the long-acting injectables have shifted from niche usage to mainstream care, especially in patients who have indicated sustained stable health and viral suppression [35,36]. With the increasing interest in the combination of diverse LA-ARVs and the expansion of global access, such medications are poised to change the HIV care standards positively. 

Beyond the advancements in treatment, scientific efforts have been intensified in the pursuit of an HIV cure. The search for a cure for HIV has continued to progress, with scientists exploring innovative approaches aimed at realizing an effective and functional cure, which effectively controls the virus without continuing treatment, or even a sterilizing treatment that will successfully eliminate HIV from the body. Still, CRISPR gene editing, which entails the precision targeting of HIV, has turned out as one of the most promising tools for fighting HIV in recent times [37]. Through precision targeting and excision of HIV DNA from the infected cells, CRISPR has the potential to eliminate HIV at the source [37]. For instance, EBT-101 by Excision BioTherapeutics (Watertown, MA, USA) is one of the CRISPR-based therapies that have undergone fast-tracked FDA designation and is currently undergoing clinical trials to assess its safety and efficacy [37]. Consequently, latency-reversing agents that seek to awaken dormant HIV are another notable and latest treatment approach currently under exploration. Presently, a key challenge in finding a cure for HIV entails the existence of latent reservoirs, where the virus not only hides but also remains inactive [38]. Thus, the aim of latency-reversing agents (LRAs) is to “shock” such latent viruses into an active state, thereby making them vulnerable to antiretroviral therapy and immune clearance [38]. Several studies have discussed the combination of LRAs and immune-based treatments to improve the clearance of reactivated HIV-infected cells [38,39]. Similarly, novel immunotherapies that enhance the body’s defense have been developed to reinforce the ability of the body’s immune system to fight HIV. Notably, the IMC-M113V therapy utilizes a T-cell receptor bispecific strategy in targeting and eliminating HIV-infected cells [38]. The initial clinical trials have demonstrated that the approach can significantly reduce the viral reservoirs, giving hope for the attainment of an effective and functional cure [38,39]. Such advancements highlight the complex approach to treating and curing HIV, combining pharmacological, immunological, and genetic strategies. 

Furthermore, advancements in vaccine technologies are opening novel prospects for HIV prevention and treatment. The mRNA and new drug delivery technologies, particularly the successful development and use of mRNA vaccines in fighting COVID-19, have resulted in immense interest in utilizing the platform for HIV prevention and treatment [3]. As a result, various trials are ongoing to test mRNA-based HIV vaccines that have been designed to stimulate immune responses against various HIV strains. For instance, NIH and Moderna have collaborated in the development of the mRNA-1644 vaccine candidate that is currently undergoing clinical trials and has demonstrated significant potential in generating broadly neutralizing antibodies [3]. These developments in HIV medication management and treatments indicate the shift towards greater accessibility of effective treatment and drugs, as well as the need for patient-centered care, making way for the development of treatments and medications that are not only effective but additionally manageable, discreet, and accessible. Beyond the evolution of HIV medication, several other aspects of HIV care have also advanced. For example, some researchers like McMahon and his team noted that various obstacles previously hindered access to HIV treatment in the United States, with limited insurance coverage being a major issue that especially affects uninsured or underinsured populations. The stigma associated with HIV remains a significant barrier, preventing individuals from seeking treatment or disclosing their status. Minority communities, especially Black and Latino populations, consistently face disparities in healthcare access and outcomes. Geographic challenges in rural areas limit access to HIV-related services and providers. Structural and social barriers also restrict the effectiveness of preventative measures like PrEP. McMahon and colleagues highlight that overcoming these challenges is crucial for improving usage and adherence among high-risk populations [6]. To tackle these barriers, the United States has implemented several targeted strategies. Expanded Medicaid programs and the Ryan White HIV/AIDS Program offer vital support to uninsured and underinsured individuals, enhancing access to antiretroviral therapy and routine care. Telehealth services and mobile clinics have helped close gaps in rural and underserved regions. Public health campaigns and community organizations work to reduce HIV-related stigma and raise awareness, particularly in minority communities. Furthermore, initiatives like Ready, Set, PrEP and the increased availability of long-acting injectable PrEP aim to boost uptake and adherence among high-risk groups [6,24,30,31].

## Conclusions

Conclusively, this systematic review evaluated recent trends in HIV/AIDS medication management. The findings show that advances in pharmacology, clinical care, and biology have led to the development of over 30 antiretroviral agents, turning HIV from a deadly virus to one that is highly treatable. Treatment has evolved from early, toxic monotherapies like AZT to more recent single-tablet regimens and long-acting injectables, which provide improved safety, adherence, and convenience. However, despite these breakthroughs, HIV/AIDS remains a major challenge. Eradicating HIV remains difficult due to the persistence of the virus in latent reservoirs, but promising interventions like latency-reversing agents are being explored. When viral load remains undetectable, sexual transmission risk is extremely low. Finally, the experience gained through decades of HIV research has informed treatment approaches for other diseases, including Ebola and some cancers.
